# Effects of intra-articular SHINBARO treatment on monosodium iodoacetate-induced osteoarthritis in rats

**DOI:** 10.1186/s13020-016-0089-6

**Published:** 2016-04-11

**Authors:** Won Kyung Kim, Hwa-Jin Chung, Yuna Pyee, Tae Jun Choi, Hyen Joo Park, Ji-Young Hong, Joon-Shik Shin, Jin Ho Lee, In-Hyuk Ha, Sang Kook Lee

**Affiliations:** College of Pharmacy, Seoul National University, Seoul, 151-742 Republic of Korea; Jaseng Spine and Joint Research Institute, Jaseng Medical Foundation, Seoul, 135-896 Republic of Korea

**Keywords:** SHINBARO pharmacopuncture, Monosodium iodoacetate-induced osteoarthritis, Intra-articular administration

## Abstract

**Background:**

SHINBARO is a refined herbal formulation used to treat inflamed lesions and bone diseases. This study aimed to investigate the anti-osteoarthritic activities of intra-articular administration of SHINBARO and determine its underlying molecular mechanism in a monosodium iodoacetate (MIA)-induced osteoarthritis rat model.

**Methods:**

Male Sprague–Dawley rats received a single intra-articular injection of MIA into the infrapatellar ligament of the right knee. Subsequently, the rats were treated with normal saline, SHINBARO, and diclofenac once daily for 21 days. Rats treated with normal saline, but not MIA, comprised the control group. Histological changes in the femur of the MIA-induced osteoarthritis rat model were observed by micro-computed tomography scanning and staining with hematoxylin and eosin, and safranin-O fast green. Serum levels of PGE_2_ and anti-type II collagen antibodies in the MIA-induced osteoarthritis rat model were measured using commercial kits. Protein levels of inflammatory enzymes (iNOS, COX-2), pro-inflammatory cytokines (TNF-α, IL-1β), and inflammatory mediators (NF-κB, IκB) in cartilaginous tissues were determined by western blot analysis.

**Results:**

Intra-articular administration of SHINBARO (IAS) at 20 mg/kg remarkably restrained the decrease in bone volume/total volume, being 28 % (*P* = 0.0001) higher than that in the vehicle-treated MIA group. IAS (2, 10, and 20 mg/kg) treatment significantly recovered the mean number of objects values with increased percentage changes of 13.5 % (*P* = 0.147), 27.5 % (*P* = 0.028), and 44.5 % (*P* = 0.031), respectively, compared with the vehicle-treated MIA group. The serum level of PGE_2_ in the IAS group at 20 mg/kg was markedly inhibited by 60.6 % (*P* = 0.0007) compared with the vehicle-treated MIA group, and the anti-collagen type II antibody level in the IAS group was reduced in a dose-dependent manner. IAS (20 mg/kg) effectively suppressed the induction of inflammation-mediated enzymes (iNOS and COX-2) and pro-inflammatory cytokines (TNF-α and IL-1β). IAS treatment also downregulated the NF-κB level and increased the IκB-α level in the MIA- induced osteoarthritis rat model.

**Conclusion:**

SHINBARO inhibited PGE_2_ and anti-type II collagen antibody production and modulated the balance of inflammatory enzymes, mediators, and cytokines in the MIA-induced osteoarthritis rat model.

**Electronic supplementary material:**

The online version of this article (doi:10.1186/s13020-016-0089-6) contains supplementary material, which is available to authorized users.

## Background

Osteoarthritis (OA) is a degenerative joint disease characterized by abrasion of articular cartilage and trabecular bone loss [1]. The chance of developing the disease increases with age, knee joint lesions, obesity, infectious diseases, and various joint inflammations [2–5]. Several previous studies suggested that abrasion of articular cartilage, subchondral bone sclerosis, and excessive formation of trabecular bone might cause OA [6–8]. Other studies highlighted the importance of prostaglandins in OA treatment [9]. Prostaglandins are enhanced by stimuli, such as lipopolysaccharide, sodium nitroprusside, or physical injury associated with OA [10], increased macrophage migration into the synovial fluid, activated nuclear factor (NF)-κB as an inflammatory transcription factor, and both cyclooxygenase (COX)-2 and inducible nitric oxide synthase (iNOS) as inflammatory enzymes [11, 12].

Regarding alleviation of OA, although non-steroidal anti-inflammatory drugs including acetaminophen are mainly prescribed during the early stage of OA, these drugs do not effectively prevent the progression of OA [13] and there have been some problems with side effects involving the gastrointestinal tract and kidneys. In particular, the use of COX-2 inhibitors is restrictively allowed because of significant side effects on cardiovascular function [14].

Monosodium iodoacetate (MIA) is an inhibitor of glyceraldehyde-3-phosphate dehydrogenase (GAPDH) that results in a reduction of glycolysis and causes articular cartilage changes related to the histological and morphological features of OA by impeding integration of the chondral structure and inducing cell death of chondrocytes [15]. Furthermore, MIA causes a repeated progression of synovial hyperplasia and inflammatory cell infiltration, destroys articular cartilage, and induces bone loss and chondral deformation [16]. Injection of MIA into the knee joints of rats is considered a suitable model that resembles the phenomena observed in human OA and is thus applied for evaluation of chondroprotective activity [17].

SHINBARO is a purified herbal formulation containing six traditional medicines, *Ledebouriellae* Radix (*Fang Feng*), *Achyranthis* Radix (*Huai Niu Xi*), *Acanthopanacis* Cortex (*Wu Jia Pi*), *Cibotii* Rhizoma (*Gou Ji*), *Glycine* Semen (*Hei Dou*), and *Eucommiae* Cortex (*Du Zhong*), that has been used to treat inflamed lesions and bone diseases [18]. Pharmacopuncture is a new form of therapy that involves both herbal medicine administration and acupuncture to manage blood extravasation and improve blood flow. Therefore, pharmacopuncturology is considered to be a medical practice in oriental medicine that involves administration of refined herbal extracts into acupoints [19]. The anti-inflammatory activity of oral SHINBARO treatment was demonstrated in an adjuvant-induced OA rat model [18]. However, the anti-arthritic activity of SHINBARO pharmacopuncture and its underlying mechanism have not yet been determined.

This study aimed to investigate the anti-osteoarthritic activities of intra-articular administration of SHINBARO and determine its underlying molecular mechanism in an MIA-induced OA rat model.

## Methods

### Preparation and composition of SHINBARO

SHINBARO was prepared by Hanpoong Pharmaceutical Co. Ltd. (Jeonju, Republic of Korea). The mixture of six crude drugs, *Ledebouriellae* Radix (4.444 g), *Achyranthis* Radix (4.444 g), *Acanthopanacis* Cortex (4.444 g), *Cibotii* Rhizoma (2.778 g), *Glycine* Semen (2.778 g), and *Eucommiae* Cortex (1.389 g), was powdered and boiled for 3 h in distilled water (1 L). The mixture was then subjected to ultrafiltration through Whatman grade 2 qualitative filter paper (GE Healthcare Life Sciences, Marlborough, MA, USA) to exclude components with molecular weights above 10,000. The resulting filtrate was lyophilized to a powder using a rotary evaporator (Eyela, Miyagi, Japan), and stored at 4 °C until use. SHINBARO was administered intra-articularly at a dose of 2, 10, or 20 mg/kg in saline and orally at a dose of 20 or 200 mg/kg in saline. The same volume of saline was used as a vehicle in control rats. The validation of SHINBARO was performed by high-performance liquid chromatography (Waters™ 600 s controller, 626 pump, temperature control module, in-line degasser, 717 plus autosampler, and 996 photodiode array detector; Waters, Bedford, MA, USA) analysis of each ingredient extract using the following six indicator biological components [18]: cimifugin for *Ledebouriellae* Radix; 20-hydroxyecdysone (0.311–0.312 mg/g) for *Achyranthis* Radix; acanthoside D (0.577–0.578 mg/g) for *Acanthopanacis* Cortex; onitin-4-O-β-D-glucopyranoside for *Cibotii* Rhizoma; genistin (0.0426–0.0427 mg/g) for *Glycine* Semen; and geniposide (0.431–0.432 mg/g) for *Eucommiae* Cortex. SHINBARO was further standardized for quality control according to the regulations imposed by the Korea Food and Drug Administration.

### Animals

Male Sprague–Dawley rats (200–220 g) were obtained from Central Laboratory Animal Inc. (Seoul, Korea), and housed in solid-bottom cages with free access to food and water. The temperature was maintained to 22 ± 2 °C, and a 12-h/12-h light/dark schedule was implemented. Prior to use, the animals were allowed 1 week for acclimatization within the work area environment. All animal experiments were approved by the local Animal Ethics Committee of Seoul National University (Additional file 1), and carried out in accordance with the Institutional Animal Care and Use Committee Guidelines of Seoul National University (Permission Number: SNU-120904-7) and the ARRIVE guideline (Additional files 2 and 3).

### MIA-induced OA rat model

Rats were anesthetized with diethyl ether and given a single intra-articular injection of 2.5 mg MIA (Sigma–Aldrich, St. Louis, MO, USA) into the infrapatellar ligament of the right knee [20]. MIA was dissolved in 0.9 % normal saline and administered in a 25-µL volume. The rats were arbitrarily divided into eight groups containing six rats each. Subsequently, the rats were treated with normal saline (vehicle-treated MIA group), 2, 10, or 20 mg/kg of SHINBARO by intra-articular administration (intra-articular SHINBARO group; IAS group), 20 or 200 mg/kg of SHINBARO by oral administration (oral SHINBARO group; OS group), and 5 mg/kg of diclofenac by oral administration (diclofenac group) once daily for 21 days. Rats treated with normal saline, and not MIA, were used as a control group (Table 1). The SHINBARO concentrations and MIA injection volume were selected based on previous evaluations [21]. After 21 days of treatment, the animals were euthanized and blood samples were collected for serum isolation. The femurs were dissected and stripped of soft tissue for analysis of the trabecular microarchitecture.

**Table 1:** Effect of SHINBARO on change in body weight of MIA-induced OA rat model

Days after treatment	Control	MIA						
		Vehicle	Intra-articular SHINBARO (mg/kg)			Oral SHINBARO (mg/kg)		Diclofenac (5 mg/kg)
			2	10	20	20	200	
0th	305.5 ± 7.9	308.1 ± 20.4	301.2 ± 17.1	305.5 ± 11.8	306.2 ± 11.8	306.2 ± 14.5	306.0 ± 16.2	288.0 ± 13.9
7th	352.9 ± 11.6	347.6 ± 14.2	335.0 ± 14.5	352.2 ± 15.0	347.2 ± 10.7	347.3 ± 11.7	351.2 ± 12.7	317.8 ± 32.0
14th	384.5 ± 9.5	378.6 ± 17.1	365.2 ± 16.1	387.8 ± 14.1	379.3 ± 11.5	376.2 ± 11.4	387.0 ± 11.7	356.0 ± 15.5
21st	411.5 ± 15.2	402.0 ± 16.3	385.5 ± 19.7	411.5 ± 15.5	401.3 ± 12.0	395.8 ± 17.3	407.8 ± 13.1	388.5 ± 9.05

SHINBARO or diclofenac was treated daily for 21 days after 2 weeks of OA induction by intra-articular injection of MIA. Body weight was measured once every 7 days after treatment of samples. Data represent as the mean ± SD (n = 6)

### Morphological analysis of bone loss

The bone microarchitecture of the femur in the region at 0.6–2.1 mm from the growth plate was scanned using a micro-computed tomography (micro-CT) system (SkyScan 1076; SkyScan, Aartselaar, Belgium). The X-ray source was set at a voltage of 50 kV and a current of 200 µA and filtered with a 0.5-mm aluminum filter. The scanning angular rotation was 180° with an angular step of 0.5°. The voxel size was fixed at 8.9 µm. The morphometric indices of the bone region were determined from the micro-CT data on 3D images using CTan software (SkyScan 1076; SkyScan, Aartselaar, Belgium). The following measures characterizing the three-dimensional structure of the trabecular bone were determined: bone volume of interest (BV; µm^3^); bone volume fraction (bone volume/total volume (BV/TV); %); and mean number of objects per slice (Obj.N). BV indicates the total volume of extracted cartilage from the same parts in each group. The ratio of BV/TV is the segmented trabecular bone volume to total tissue volume. Obj.N implies the connectivity of the specimens, which consisted of several cross-sectional slices of cartilage in a three-dimensional structure.

### Histopathological analysis

The right knee joints from the tibia to the distal metatarsal including the tarsal joint were resected and fixed with 10 % neutral-buffered formalin for 24 h at 4 °C. The fixed specimens were decalcified with 20 % formic acid for 3 days and embedded in paraffin. Sections of the tissue specimens were acquired from the paraffin blocks at 5 µm thickness, deparaffinized, and rehydrated in the order of xylene, absolute alcohol, and 50 % alcohol. The rehydrated sections were stained with hematoxylin and eosin (H&E) for observation of morphological changes in the articular tissues and safranin-O fast green (SOFG) for evaluation of the proteoglycan (PG) contents.

### Measurement of prostaglandin E_2_ (PGE_2_) level

The serum PGE_2_ levels were measured by enzyme immunoassay (EIA) using a prostaglandin E metabolite EIA kit (Cayman Chemical Company, Ann Arbor, MI, USA). Briefly, aliquots of prepared standard solutions and sample sera were plated in 96-well plates from the PGE_2_ EIA kit, and a PGE_2_-acetylcholinesterase (AChE) conjugate (PGE Tracer) and PGE_2_ monoclonal antibody solution were added and allowed to react at 4 °C for 24 h. Next, Ellman’s reagent, which contains an AChE substrate for color formation, was added to the wells and the optical density was measured at 412 nm (VersaMax ELISA Microplate Reader; Molecular Devices, Sunnyvale, CA, USA). The PGE_2_ concentrations in the serum samples were determined using a four-parameter logistic equation (logit(B/B_0_) = ln[B/B_0_/(1–B/B_0_)]) by comparisons with the absorbances of the PGE_2_ standard solutions at several concentrations (8, 16, 31, 62, 125, 250, 500, and 1000 pg/mL).

### Measurement of anti-type II collagen antibody level

The serum anti-type II collagen antibody levels were assayed using a rat anti-type I and type II collagen IgG ELISA kit (Chondrex Inc., Redmond, WA, USA) in accordance with the manufacturer’s instructions.

### Ex vivo biochemical analysis of inflamed tissues

The right knee joint cartilages of the rats were removed at the end of the treatment period. The tissues were homogenized using Nuclear Extract Kit (Active Motif, Carlsbad, CA, USA) in accordance with the manufacturer’s instructions. The protein levels of inflammatory enzymes (iNOS, COX-2), pro-inflammatory cytokines (tumor necrosis factor (TNF)-α, interleukin (IL)-1β), and inflammatory mediators (NF-κB, nuclear factor of κ light polypeptide gene enhancer in B-cells inhibitor (IκB)) in the cartilaginous tissues were determined by western blot analysis.

### Western blot analysis

The proteins in the rat articular cartilage samples were resolved by 6–15 % sodium dodecyl sulfate–polyacrylamide gel electrophoresis (SDS-PAGE) and transferred onto PVDF membranes (Millipore, Bedford, MA, USA). The membranes were blocked with blocking buffer, comprising 5 % bovine serum albumin in phosphate-buffered saline containing 0.1 % Tween-20 (PBST), for 1 h at room temperature. After three washes with PBST, the membranes were incubated with primary antibodies against β-actin (1:1000), iNOS (1:1000), COX-2 (1:1000), IL-1β (1:1000), NF-κB (1:500), IκB-α (1:200) (Santa Cruz Biotechnology, Santa Cruz, CA, USA) and TNF-α (1:1000) (Cell Signaling, Danvers, MA, USA) diluted in 2.5 % bovine serum albumin overnight at 4 °C. The membranes were washed three times with PBST and incubated with corresponding secondary antibodies diluted in PBST (1:1000) for 2 h at room temperature. After three washes with PBST, the membranes were visualized with a WEST-ZOL plus Western Blot Detection System (Intron Biotechnology, Sungnam, Korea), and analyzed using an LAS 4000 system (Fuji Film Corp., Tokyo, Japan).

### Statistical analysis

All experiments were repeated at least three times. Data were presented as means ± standard derivation for the indicated numbers of independently performed experiments. Statistical significance was assessed by one-way analysis of variance coupled with a Dunnett’s *t* test. Values of *P* < 0.05 were considered statistically significant. All statistical analyses were performed using Graph Pad Prism 5.0 for Windows (GraphPad Software, La Jolla, CA, USA).

## Results

### Body weight changes

Changes in body weight were monitored during administration of SHINBARO or diclofenac and measured on days 0, 7, 14, and 21 as evaluation parameters for the courses of stress and toxicity in the animals. The body weight in the vehicle-treated MIA group increased at a similar rate to that in the control group (Table 1). In addition, the IAS group, OS group, and diclofenac group did not show any significant differences compared with the vehicle-treated MIA groups. No overt toxicity was observed in the IAS and OS groups compared with the vehicle-treated control groups.

### Morphological analysis of bone loss

**Fig. 1:**
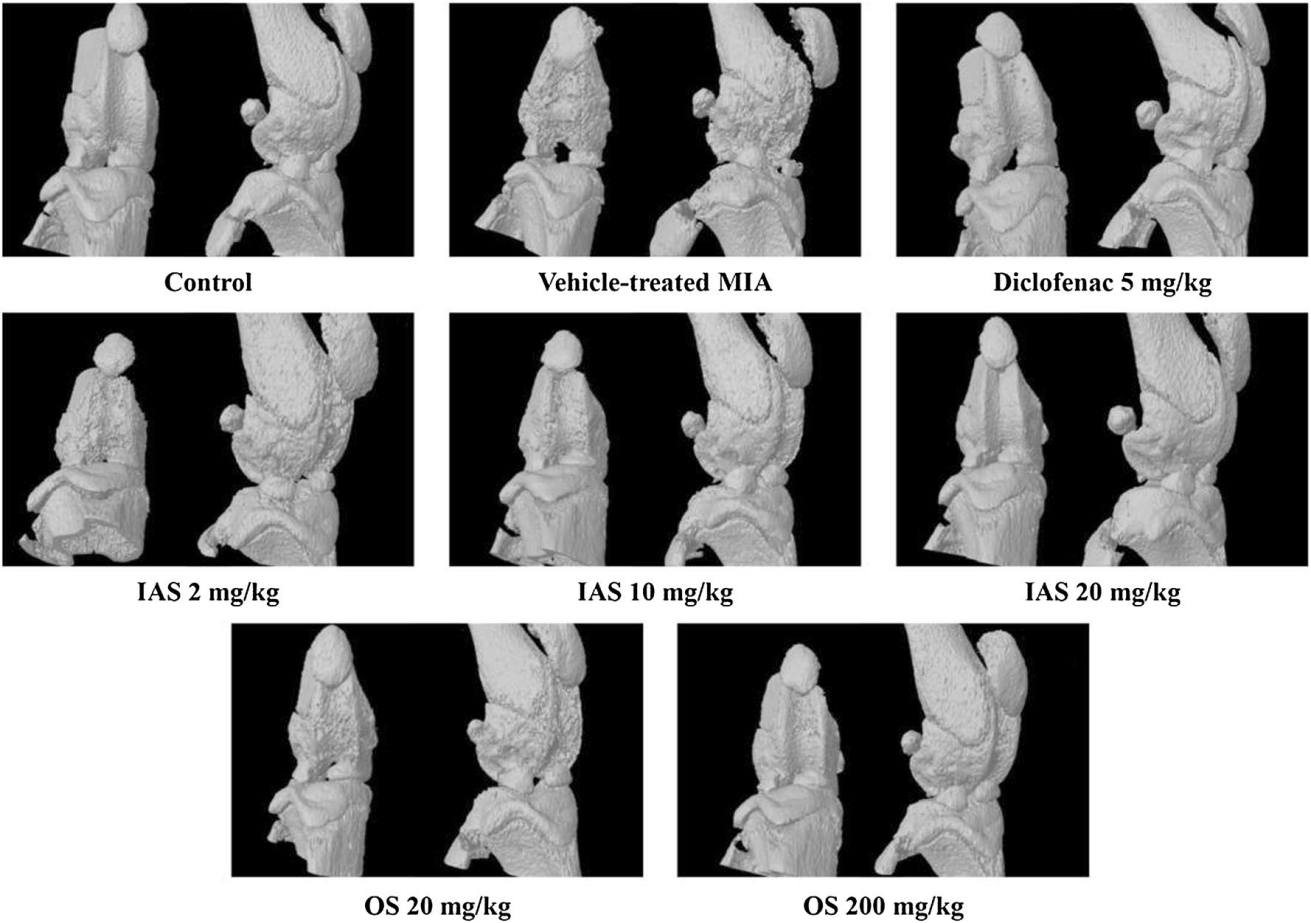
Roentgenograph analysis of the knee joint after treatment of SHINBARO in MIA-induced OA rat model. Micro-cartilage and patellae of the normal and experimental animals were scanned with microCT (Skyscan1076). Data represent as the mean ± SD (n = 6). *IAS* intra-articular SHINBARO, *OS* oral SHINBARO

**Fig. 2:**
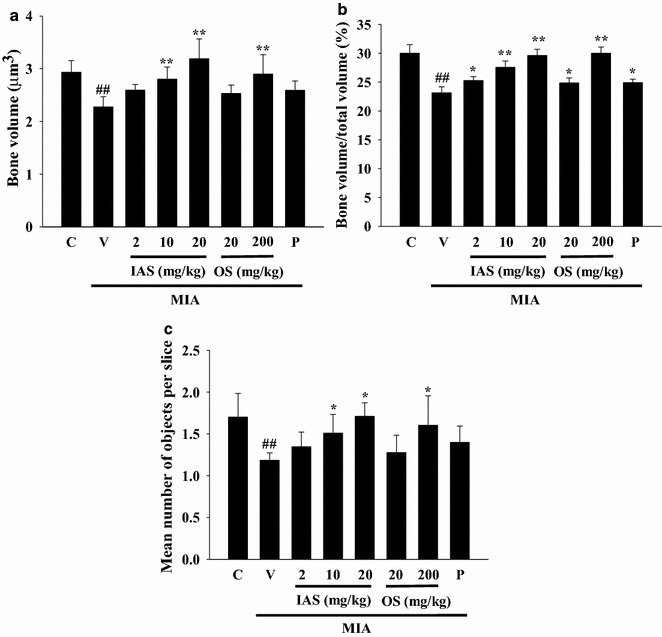
Effect of SHINBARO on the bone morphometric parameters in MIA-induced OA rat model. The bone morphometric parameters including BV (**a**), BV/TV (**b**), and Obj.N (**c**) were analyzed with micro-CT SkyScan CTAN software. Data represent the mean ± SD (n = 6). *P < 0.05 and **P < 0.01 indicate statistically significant differences from the vehicle-treated MIA group. ^##^ *P* < 0.01 indicates statistically significant differences from the control group. *C* control group, *V* vehicle-treated MIA group, *IAS* intra-articular SHINBARO group, *OS* oral SHINBARO group, *P* positive control group (Diclofenac 5 mg/kg)

The destruction of the trabecular bone in the femur was observed by three-dimensional micro-CT to determine the effects of SHINBARO on the trabecular bone microarchitecture. As shown in Fig. 1, MIA caused significant deterioration of the trabecular bone architecture compared with the control group. However, IAS or OS treatment delayed or recovered the destruction of the trabecular bone in the MIA-induced OA model. Protective activity was also observed for oral treatment of diclofenac, a positive control, under the same experimental conditions. In addition, the bone microstructural index parameters were measured using NRrecon software (Fig. 2). The BV of the trabecular bone in the femur was clearly decreased in the vehicle-treated MIA group by 22.4 % (*P* = 0.0042) compared with the control group. However, IAS (20 mg/kg) treatment remarkably restrained the decrease in BV (*P* = 0.0045), being 40 % higher than that in the vehicle-treated MIA group. The BV of the trabecular bone in the femur was alleviated by IAS in a dose-dependent manner and recovered to almost the same level as the control group. Diclofenac treatment insignificantly increased the BV of the trabecular bone in the femur by 13.8 % in the MIA-induced OA model (*P* = 0.053). In addition, BV/TV was decreased in the vehicle-treated MIA group (22.9 %, *P* = 0.0089) compared with the control group. IAS (2, 10, and 20 mg/kg) treatment effectively recovered the BV/TV values in a dose-dependent manner with increased percentages of 9.2 % (*P* = 0.015), 19.2 % (*P* = 0.001), and 28.1 % (*P* = 0.0001), respectively, compared with the vehicle-treated MIA group, while diclofenac exhibited an increase of 7.7 % in BV/TV. Obj.N was markedly reduced in the vehicle-treated MIA group (30.4 %, *P* = 0.0063) compared with the control group, and IAS (2, 10, and 20 mg/kg) treatment significantly recovered the Obj.N values with increased percentage changes of 13.5 % (*P* = 0.147), 27.5 % (*P* = 0.028), and 44.5 % (*P* = 0.031), respectively, compared with the vehicle-treated MIA group. The diclofenac group also exhibited recovery of Obj.N with an increased percentage change of 18.0 % (*P* = 0.095) compared with the vehicle-treated MIA group. OS (20 or 200 mg/kg) treatment also improved the damage to the cartilage with increased percentage changes of 7.7 % (*P* = 0.405) and 35.3 % (*P* = 0.013), respectively, but the activity was relatively low compared with that of IAS treatment.

### Histopathological analysis

**Fig. 3:**
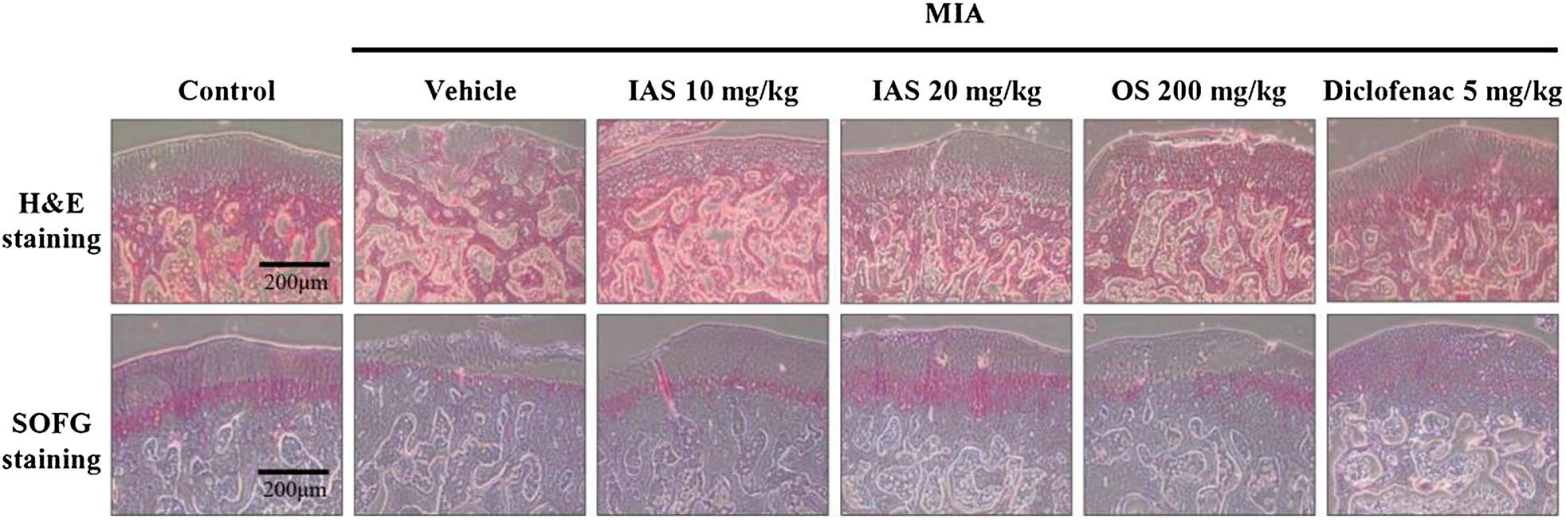
Histopathological analysis of the knee joint tissues after treatment of SHINBARO in MIA-induced OA rat model. The knee joint tissues from MIA-induced OA rat model which was treated with vehicle, SHINBARO or diclofenac were stained with hematoxylin and eosin (H&E) and Safranin O-fast green (SOFG) (magnification ×100)

We performed H&E staining for the articular cartilage surfaces of the femoral condyle and tibial plateau to determine whether IAS treatment restored the damaged surface of the knee joint toward healing. The vehicle-treated MIA group revealed severe irregular abrasions with rough edges around the femur and tibia, indicative of bone lysis, swelling, and tendency for patellar displacement (Fig. 3). This damage was significantly attenuated with the appearance of smoother articular cartilage surfaces by IAS (20 mg/kg) treatment. MIA-induced articular cartilage damage was also restored in the positive control group treated with diclofenac. SOFG staining, which stains PGs and is indicative of the degree of degeneration of cartilaginous tissue [22], was performed to observe the changes in morphology of the cartilaginous tissues. The SOFG-stained control group exhibited normal cartilage PG staining, while the vehicle-treated MIA group revealed severely damaged cartilage with marked fibrillation and PG depletion. Treatment with SHINBARO or diclofenac significantly suppressed the MIA-induced loss of PG and prevented cartilage damage (Fig. 3).

### Analysis of biomarkers in serum

**Fig. 4:**
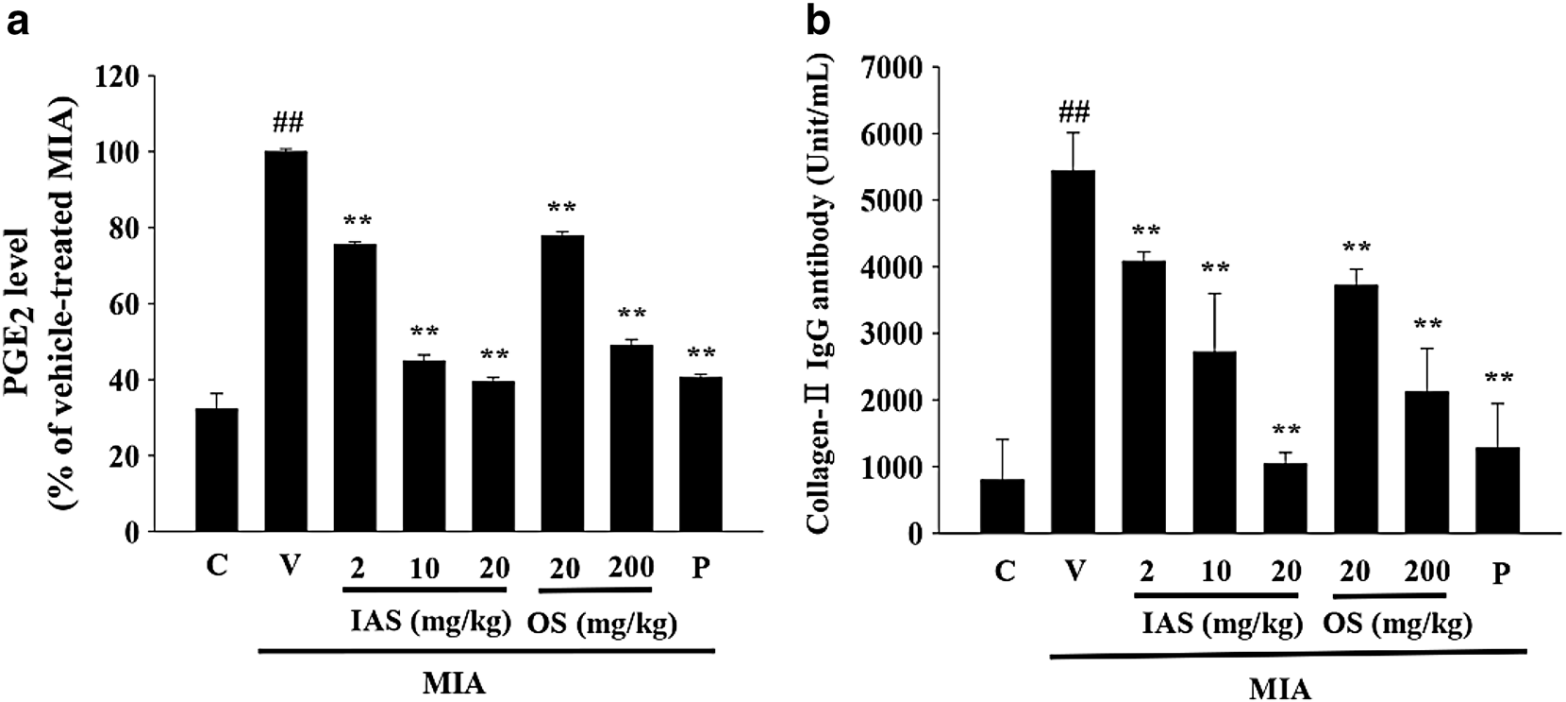
Effect of SHINBARO on the serum levels of prostaglandin E_2_ and collage-II antibody in MIA-induced OA rat model. Data represent the mean ± SD (n = 6). *P < 0.05 and **P < 0.01 indicate statistically significant differences from the vehicle-treated MIA group. ^##^ *P* < 0.01 indicates statistically significant differences from the control group. *C* control group, *V* vehicle-treated MIA group, *IAS* intra-articular SHINBARO group, *OS* oral SHINBARO group, *P* positive control group (Diclofenac 5 mg/kg)

The effects of IAS treatment on inflammatory biomarkers were determined using serum prepared from blood samples collected in the MIA-induced OA rat model. The serum PGE_2_ level in the MIA-treated group was significantly increased by 67.8 % (*P* = 0.0045) compared with the control group (Fig. 4a). IAS (20 mg/kg) treatment markedly inhibited the elevated serum PGE_2_ level by 60.6 % (*P* = 0.0007) compared with the vehicle-treated MIA group. The serum PGE_2_ level in the diclofenac group was inhibited by 59.5 % (*P* = 0.0012) under the same experimental conditions. Furthermore, the serum level of anti-type II collagen antibodies, which are responsible for activation of oxidative stress with the formation of peroxynitrite (ONOO^−^) [7], was increased in the MIA-treated group, while IAS treatment significantly decreased the anti-type II collagen antibody level in a dose-dependent manner (Fig. 4b).

### Analysis of biomarkers in cartilaginous tissue

**Fig. 5:**
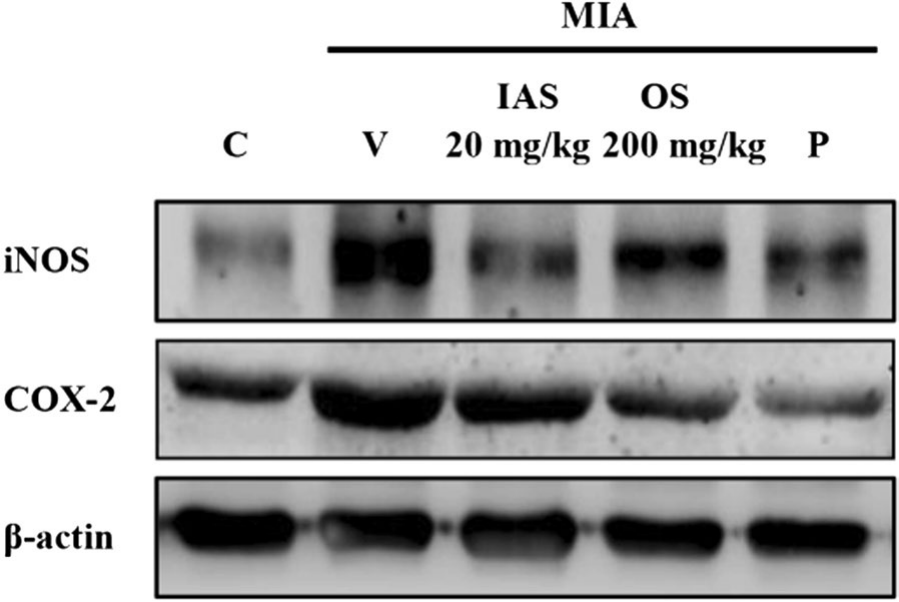
Effect of SHINBARO on the protein levels of iNOS and COX-2 in cartilage from the knee joint after treatment of SHINBARO in MIA-induced OA rat model. The expressions of iNOS and COX-2 in the extracted cartilage tissues were determined by western blot analysis as described in the “Methods” section. Data were representative of three separate experiments. β-actin was used as an internal standard. *C* control group, *V* vehicle-treated MIA group, *IAS* intra-articular SHINBARO group, *OS* oral SHINBARO group, *P* positive control group (Diclofenac 5 mg/kg)

The effects of IAS treatment on the protein expression levels of inflammation-mediated enzymes, such as iNOS and COX-2, were determined in the MIA-induced OA rat model to determine the mechanism of action of IAS treatment on the inhibition of inflammatory biomarker production. Overproduction of NO and PGE_2_ is concomitant with overexpression of iNOS and COX-2, leading to swelling, irritation, and pain [23]. MIA treatment induced overexpression of iNOS and COX-2 in the cartilaginous tissues, while IAS (20 mg/kg) or OS (200 mg/kg) treatment effectively suppressed the induction of iNOS and COX-2 expression (Fig. 5).

**Fig. 6:**
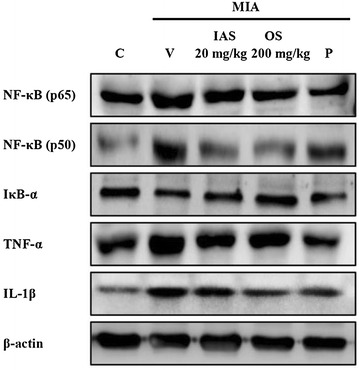
Effect of SHINBARO on the protein levels of NF-κB, IκB-α, TNF-α and IL-1β in cartilage from the knee joint after treatment of SHINBARO in MIA-induced OA rat model. The expressions of NF-κB, IκB-α, TNF-α and IL-1β in the extracted cartilage tissues were determined by Western blot analysis as described in the “Methods” section. Data were representative of three separate experiments. β-actin was used as an internal standard. *C* control group, *V* vehicle-treated MIA group, *IAS* intra-articular SHINBARO group, *OS* oral SHINBARO group, *P* positive control group (Diclofenac 5 mg/kg)

iNOS and COX-2 expression are regulated by NF-κB [24]. NF-κB is composed of p65/p50 subunits and exists in the cytosol as inactive heterodimers bound to the inhibitory protein IκB-α. IκB-α stimulated by pro-inflammatory signals is phosphorylated by IκB kinase and degraded by a 26S proteasome-mediated pathway to facilitate translocation of NF-κB into the nucleus and regulate the transcription of iNOS and COX-2 [25]. MIA treatment upregulated the nuclear protein levels of the NF-κB subunits (p65 and p50), while IAS treatment suppressed these biomarker expression levels, especially that of the p50 subunit (Fig. 6). In addition, the protein expression of IκB-α was downregulated in the vehicle-treated MIA group compared with the control group, while IAS treatment increased the IκB-α level in the MIA-induced OA rat model. IAS treatment might inhibit the inflammatory responses through suppression of MIA-induced NF-κB activation.

Further studies were conducted to correlate the anti-inflammatory effects of IAS treatment with the regulation of expression of pro-inflammatory cytokines. The effects of IAS treatment on the expression levels of these pro-inflammatory cytokines were investigated in the MIA-induced OA rat model. The protein levels of TNF-α and IL-1β revealed overexpression in the MIA-treated group compared with the control group, while IAS treatment suppressed the elevated expression levels of TNF-α and IL-1β in the cartilaginous tissues of the MIA-induced OA rat model (Fig. 6). Therefore, IAS treatment inhibited the inflammatory response through blockade of the expression of pro-inflammatory cytokines, such as TNF-α and IL-1β.

## Discussion

OA is a degenerative knee joint disease that causes intense pain and functional disability [26]. The disease is classified into two categories: primary or idiopathic arthritis that involves endocrinologic, genetic, and nutritional factors and secondary or subsequent arthritis that is incurred by injury, disorder, and malformation capable of causing damage to the articular cartilage [27, 28].

Although anti-inflammatory effects of OS treatment were previously reported [29], the pharmacological effects of IAS treatment, which is already practiced in clinical settings, and its underlying mechanisms of action remain to be determined. Therefore, the present study was conducted to investigate the anti-inflammatory activity of IAS treatment and its underlying mechanisms of action in the MIA-induced OA rat model.

The MIA used in the present study inhibits articular cartilage structural incorporation through suppression of GAPDH activity in the cartilage, leading to histopathological and pathomorphologic changes in the articular cartilage [30]. SHINBARO prevented the degeneration of the trabecular bone microarchitecture in the distal femur of the rat. IAS treatment also markedly restored bone loss in the MIA-induced OA model (Fig. 1). These data were supported by the protective effect of IAS treatment on the bone morphometric parameters (Fig. 2). In addition, no significant body changes or obvious toxicity were found in the IAS group (Table 1). These results indicated that IAS treatment not only preserves the bone mass, but also recovers the bone microstructural deterioration associated with MIA-induced OA without toxicity.

Healthy cartilage tends to maintain a balance between synthesis and degradation of extracellular matrix (ECM) components, such as PGs and type II collagen. However, the decomposition of the ECM becomes increased in inflamed cartilage, leading to an off-balance of matrix production and damage to the cartilaginous tissues [31]. The H&E and SOFG staining in the vehicle-treated MIA group revealed significant PG loss and lesion development, and confirmed that the cartilage injury was attenuated by IAS treatment (Fig. 3). In addition, IAS treatment markedly reduced the elevated serum level of anti-type II collagen antibodies associated with MIA in a dose-dependent manner (Fig. 4b). These findings indicate that IAS treatment might delay bone loss through decreased bone turnover.

Major biomarkers for inflammation responses were determined in serum to further confirm the effects of IAS treatment on the regulation of inflammatory responses. PGE_2_ acts as an important inflammatory mediator and is a major product of COX activity, which functions pathologically in inflammatory, autoimmune, and neoplastic diseases [32]. In this study, the serum PGE_2_ level was significantly higher in the vehicle-treated MIA group compared with the control group, while IAS treatment inhibited the elevation of PGE_2_ production in the MIA-induced OA rat model (Fig. 4a).

Overproduction of PGE_2_ is highly associated with overexpression of iNOS and COX-2 in inflammatory responses [33]. The effects of IAS on iNOS and COX-2 protein expression levels were determined to investigate the mechanism responsible for the inhibition of PGE_2_ production mediated by IAS treatment. IAS suppressed the MIA-induced overexpression of iNOS and COX-2 protein levels in a dose-dependent manner (Fig. 5).

We further examined whether IAS treatment regulates the protein expression levels of upstream mediators leading to the production of iNOS and COX-2. NF-κB exists in the cytosol of cells as an inactive heterodimer bound to the inhibitory protein IκB with Rel A (p65), c-Rel, RelB, NF-κB1 (p50/150), and NF-κB2 (p52/100) [34]. However, NF-κB becomes degraded with IκB sequestration when inflammatory cytokines, TNFs, and lymphotoxins are activated by stimuli, such as mitogens, bacteria, ultraviolet light, oxidants, tetradecanoyl phorbol-13-acetate, ionizing radiation, and phosphatase inhibitors [35–37]. The activated heterodimers of NF-κB subunits p65 and p50 in the cytosol translocate into the nucleus and bind to NF-κB-binding sites in gene promoters or enhancers, thereby inducing the transcription of downstream target genes, such as cytokines, cytokine receptors, cell adhesion molecules, and growth factors. IκB is negatively regulated by phosphorylation on serine residues, which is controlled by two IκB kinases, IKKα and IKKβ [38]. In the present study, IAS treatment downregulated the protein level of NF-κB and upregulated the protein level of IκB (Fig. 6). These findings suggest that the anti-inflammatory effects of IAS treatment are associated with inhibition of NF-κB activation.

The anti-inflammatory effects of IAS treatment were also observed by measurements of the protein expression levels of pro-inflammatory cytokines, such as TNF-α and IL-1β. These cytokines promote the catabolic processes in OA, causing cartilage degradation. MIA can drive the overexpression of pro-inflammatory cytokines, but the TNF-α and IL-1β levels were effectively reduced by IAS treatment in the MIA-induced OA model (Fig. 6). These data suggest that IAS treatment inhibits inflammatory responses by suppressing the expression of pro-inflammatory cytokines, including TNF-α and IL-1β.

## Conclusion

SHINBARO inhibited PGE_2_ and anti-type II collagen antibody production and modulated the balance of inflammatory enzymes, mediators, and cytokines in the MIA-induced OA rat model.

## Additional files

10.1186/s13020-016-0089-6 Animal liscense.
10.1186/s13020-016-0089-6 Animal care and use guidelines.
10.1186/s13020-016-0089-6 ARRIVE guideline.

## Abbreviations

MIA: monosodium iodoacetate
OA: osteoarthritis
H&E: hematoxylin and eosin
SOFG: safranin-O fast green
PGE_2_: prostaglandin E_2_
iNOS: inducible nitric oxide synthase
COX-2: cyclooxygenase-2
TNF-α: tumor necrosis factor- α
IL-1β: interlukin-1β
NF-κB: nuclear factor-κB
IκB: inhibitory protein κB
LPS: lipopolysaccharide
GADPH: glyceraldehyde-3-phosphate dehydrogenase
IAS: intra-articular SHINBARO
OS: oral SHINBARO
BV: bone volume of interest
Obj.N: mean number of objects per slice
EIA: enzyme immunoassay
AChE: acetylcholinesterase
PG: proteoglycan
NSAIDs: non-steroidal anti-inflammatory drugs
ECM: extracellular matrix
IKK: IκB kinase
