# Supplementary figures and images for: Effects of intra-articular SHINBARO treatment on monosodium iodoacetate-induced osteoarthritis in rats
Source: Chin Med. 2016 Apr 11;11:17. doi: 10.1186/s13020-016-0089-6 (PMC4827221; doi:10.1186/s13020-016-0089-6)

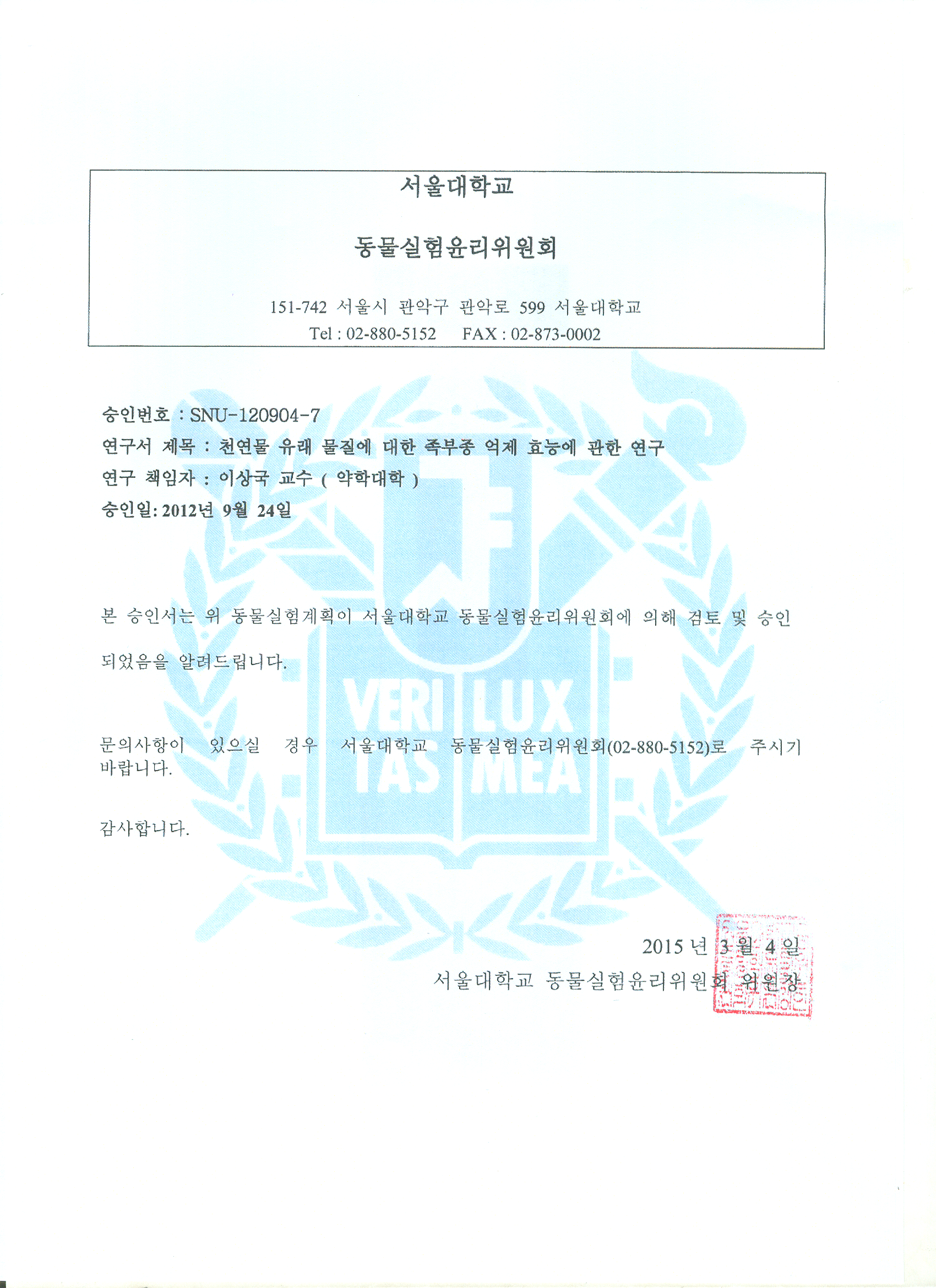

Supplement: Supplementary file 1 — 10.1186/s13020-016-0089-6 Animal liscense. [file 13020_2016_89_MOESM1_ESM.tiff]
