# Supplementary material for: Effects of intra-articular SHINBARO treatment on monosodium iodoacetate-induced osteoarthritis in rats
Source: Chin Med. 2016 Apr 11;11:17. doi: 10.1186/s13020-016-0089-6 (PMC4827221; doi:10.1186/s13020-016-0089-6)
Supplement: Supplementary file 2 — 10.1186/s13020-016-0089-6 Animal care and use guidelines. [file 13020_2016_89_MOESM2_ESM.pdf]

ANIMAL STUDY PROPOSAL

1. Submission date : 10. 09. 2012.

2. Pricipal Investigator

College and Department	Title	Name	Professor	Telephone	e-mail
College : Pharmacy<br>Department : Pharmacy	Ph.D. candidate	Won Kyung Kim	Sang Kook Lee	010-6434-6764	dnjsrud6764 @naver.com

3. Initial / Renewal / Modification Submission

	Initial submission	Already approved		
		Approval number	Expiration Date	Reason for renewal
Check(∨)	∨			

4. Information of Investigators

Name	Title	Telephone	e-mail	SOP training number[1]
Hwa Jin Jeong	Ph. D.	010-4745-8615	badduck@hanmail.net	ILAR-10-04-009

1) Check only if you get the training number of SOP.

5. Animal use

Project title	Study on effect of SHINBARO acupuncture on monosodium iodoacetate-induced osteoarthritis in rat
Study objectives	To investigate the anti-osteoarthritic effects of SHINBARO acupuncture in monosodium iodoacetate-induced osteoarthritis
Expected results	The intra-articular administration of SHINBARO is expected to heal the monosodium inodoacetate-induced osteoartheritis in dose-dependent manner
Outline of study	1. Monosodium iodoacetate-induced osteoarthritis rat model<br>- a single intra-articular injection of 2.5 mg monosodium iodoacetate into the infrapatellar ligament of the right knee<br><br>2. Treatment<br>- intra-articular administration of SHINBARO (IAS, 2, 10, and 20 mg/kg) or oral administration of SHINBARO (OS, 20 and 200 mg/kg) or oral administration of diclofenac, positive control (5 mg/kg)<br>- (n=6)*(Control, Vehicle, positive control, IAS 3 groups, OS 2 groups)<br><br>3. ex vivo study

6. Rationale for animal use

Rationale for animal use	SHINBARO has been used as traditional medicines for treatment of several inflammatory disorder and bone disease. But its underlying mechanisms of action remain to be elucidated. In this lab, in vivo osteoarthritis rat model had been already established. Basic data from this study which investigates the anti-osteoarthritic activity of SHINBARO acupuncture could be used as the evidence of clinical trial.
Appropriateness of the species selected	The monosodium iodoacetate-induced osteoarthritis rat model is known to be similar to human degenerative arthritis and the deterioration of trabecular microarchitecture is clear in this model. Therefore, the articular injection of monosodium iodoacetate into rats is appropriate to evaluate anti-osteoarthrtic activity of SHINBARO acupuncture.
Appropriateness of the number of animals to be used	The number of animals to be used (n=6) is appropriate to provide data for a more accurate assessment and to be the greatest extent possible consistent with sound scientific and statistical standards.

7. Animal Requirements

Genus	Rat	Species	Norvegicus
Strain, subspecies, or breed	SD	Common name	White laboratory rat
Age	Weight	Size	Sex (check)
6 weeks old	200 g	10-12 cm	∨♂/♀
Bacteriological status (Check)			
Germfree	Gnotobiote	∨ SPF	Conventional
Source(s) (Telephone)	Chung-Ang laboratory animal (+82-2-3461-5255)	Transporter (Telephone)	Chung-Ang laboratory animal (+82-2-3461-5255)
Number of Animals to be used			
Year 1	48	Year 2	
Year 3		Total	48
Primary housing location(s) (Telephone)		Institute of laboratory animal Seoul National Univ. (+82-2-880-5334)	

8. Description of Experimental Design and Animal procedures

1) Schedule

Location where manipulation will be conducted	The date of beginning	The date of end	Experimental period
Institute of laboratory animal Seoul National Univ.	2012-09-24	2012-10-24	30 days

2) Bio-safety Level (Check (∨)

Grade	BS I	BS II	BS III	BS IV
Check				

3) Animal Experimental Procedures

Restraint	Method	by hands	
	The number of restraint	once daily	
	The necessary time in restraining once	within 1 minute	
Animal identification methods[2]	Ear tags		
Stress[3]	None		
Experimental injections or inoculations	Infectious agents, adjuvant	SHINBARO acupuncture,<br>diclofenac (in 0.9 % normal saline)	
	Route ,volume, substances	1) intra-articular, 0.02 ml, SHINBARO<br>2) p.o., 0.2 ml, SHINBARO<br>3) p.o., 0.2 ml, diclofenac	
	Schedules	once daily	
Sampling	Type	1) articular tissue<br>2) blood	
	Volume, frequency	1) 10 mg, once<br>2) 5 ml, once	
	Methodology and point	The animals will be sacrificed and femur bones for tissue analysis and blood samples for serum isolation will be collected.	
Surgical procedures	None	Survival surgical procedures	Non-survival surgical procedures
	V		
Veterinary care	If animals suffer from diseases in procedure, they will get treatment from expert or be sacrificed in emergency.		
Experimental endpoint criteria	Sacrifice of animals will be determined by experimental endpoint criteria. But if body weights of animals are decreased by sample toxicity or skin conditions are deteriorated, they will be sacrificed directly.		

2) e.g., ear tags, tattoos, collar, cage card, implant

3) e.g., food or water deprivation, noxious stimuli, environmental stress

4) Hazardous agents[4)]

Hazardous agent	×	○	Date of Biosafety Approval[5)]	The name of the agents	Route & dose	Tracking #
Radionuclides	V					
Biological agents	V					
Hazardous Chemicals or Drugs	V					
Recombinant DNA	V					
etc.	V					

9. Animal welfare

<table>
<tr><td rowspan="3">The grade of Pain</td><td>Species</td><td>Grade</td><td colspan="5">Number of animals used each year</td></tr>
<tr><td rowspan="2"></td><td rowspan="2">D</td><td>48</td><td></td><td></td><td></td><td></td></tr>
<tr><td></td><td></td><td></td><td></td><td></td></tr>
<tr><td rowspan="3">Alternatives[6)]</td><td colspan="2">Database references</td><td colspan="5">Institute of laboratory animal resources Seoul National University</td></tr>
<tr><td colspan="2">The date of the search, keyword</td><td colspan="5">September 10, 2012 , monosodium iodoacetate-induced osteoarthritis</td></tr>
<tr><td colspan="2">Consideration for animal number reduction</td><td colspan="5">The number of animals to be used (n=6) is appropriate to provide data for a more accurate assessment and to be the greatest extent possible consistent with sound scientific and statistical standards.</td></tr>
<tr><td rowspan="2">Anesthesia/ Analgesia/ Tranquilization[7)]</td><td colspan="2">The name of the agents</td><td colspan="5">None</td></tr>
<tr><td colspan="2">Route, dose, schedule of administration)</td><td colspan="5">None</td></tr>
<tr><td>Method of euthanasia</td><td colspan="7">CO_2 inhalation</td></tr>
<tr><td>Disposition of animals</td><td colspan="7">request to institute of laboratory animal resource Seoul National University</td></tr>
</table>

4) Use of hazardous agents requires the approval of the institutional Biosafety Office/Committee.

5) Also describe the organization of biosafety approval.

6) If any procedures fall into USDA's Classification D or E

7) If any procedures fall into USDA's Classification D or E

10. IACUC assessment

Approval NO.	Disapproval reason
SNU-120904-7	
